# Supplementary figures and images for: The dynamics of centromere assembly and disassembly during quiescence
Source: bioRxiv. 2025 Sep 8:2025.09.08.674938. Preprint. [Version 1] doi: 10.1101/2025.09.08.674938 (PMC12440008; doi:10.1101/2025.09.08.674938)

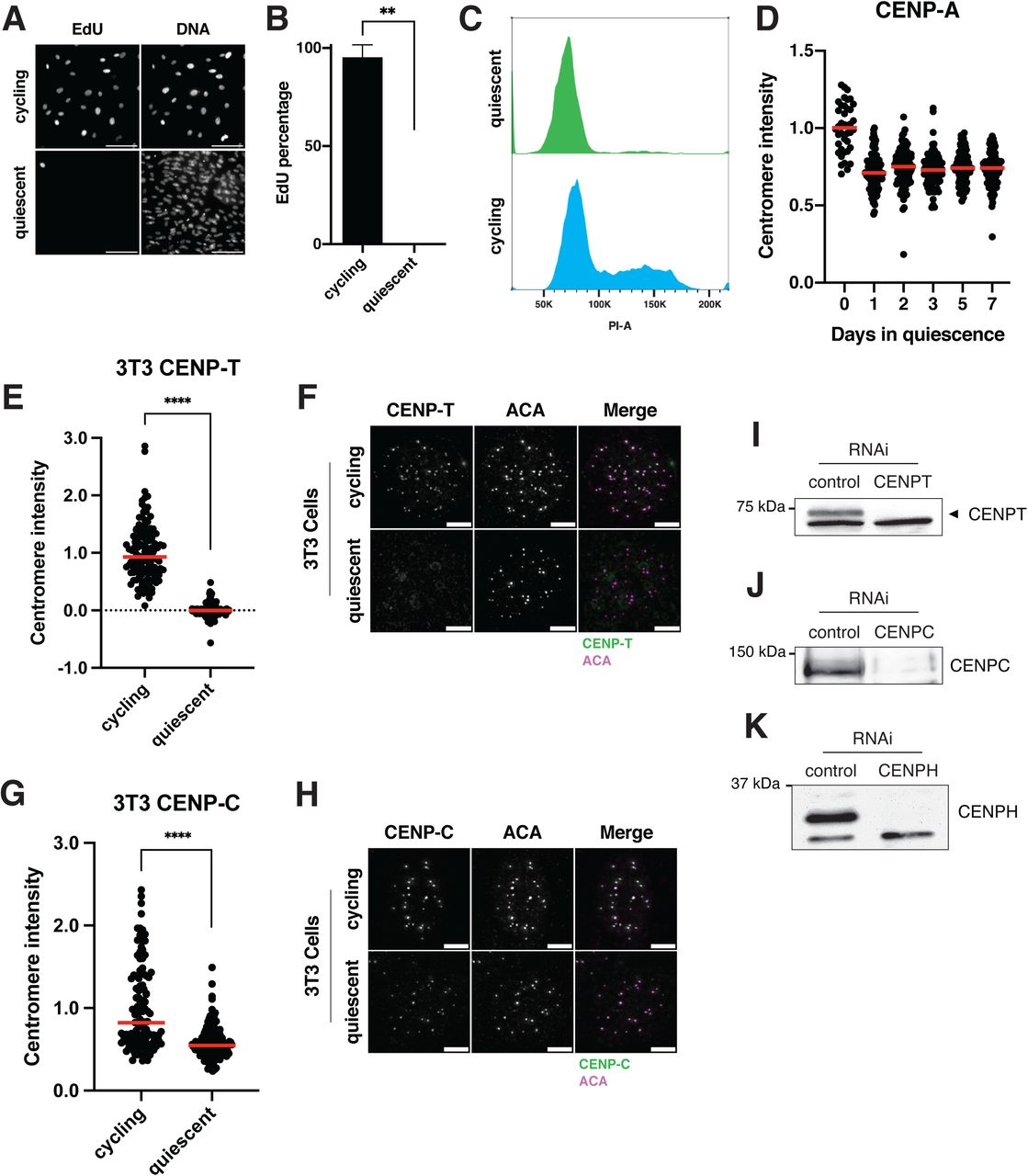

Supplement: figure-S1 — Supplementary Figure 1: Controls for quiescence induction and conservation of quiescent centromere behavior in mouse cells A. Representative images of EdU staining of cycling and quiescent cells. Cells were incubated for 48 hours in 5-ethynyl-2’ deoxyuridine (EdU), a nucleotide analog that monitors DNA replication and progression through the cell cycle. Scale bar = 100μm. B. Graph showing the percentage of EdU positive cells for the indicated condition. Cells were incubated for 48 hours in EdU. Bars represent mean ± standard deviation of three replicates. Mean of cycling is 95.12, mean of quiescence is 0.2. ** represents p = 0.0015. C. Histogram showing the distribution of propidium iodide (PI) staining for cycling cells (blue) or cells in quiescence for 7 days (green) as measured by flow cytometry. D. Graph showing CENP-A centromere intensity level over time of quiescence entry. Each point indicates the average centromere intensity level for all centromeres of a single cell, adjusted for background. Intensity values were normalized to day 0. Red line represents the median. Points were from 1 replicate, as this result has already been previously shown. n = 41, 110, 94, 84, 110, and 93 cells for 0, 1, 2, 3, 5, and 7-day time points respectively. E. Graph showing CENP-T centromere intensity levels in cycling and quiescent mouse 3T3 cells. Each point indicates the average centromere intensity level for a single cell, adjusted for background. Intensity values were normalized to cycling condition. Red line represents the median. Points were aggregated from 2 replicates. n = 121 and 123 cells for cycling and quiescent respectively. **** represents p<0.0001. F. Representative immunofluorescence images of cycling and quiescent mouse 3T3 cells. Cells were stained with mouse CENP-T and anti-centromere (ACA) antibodies. Scale bar = 5μm. G. Graph showing CENP-C centromere intensity levels in cycling and quiescent mouse 3T3 cells. Each point indicates the average centromere inte [file figure-S1.jpg]

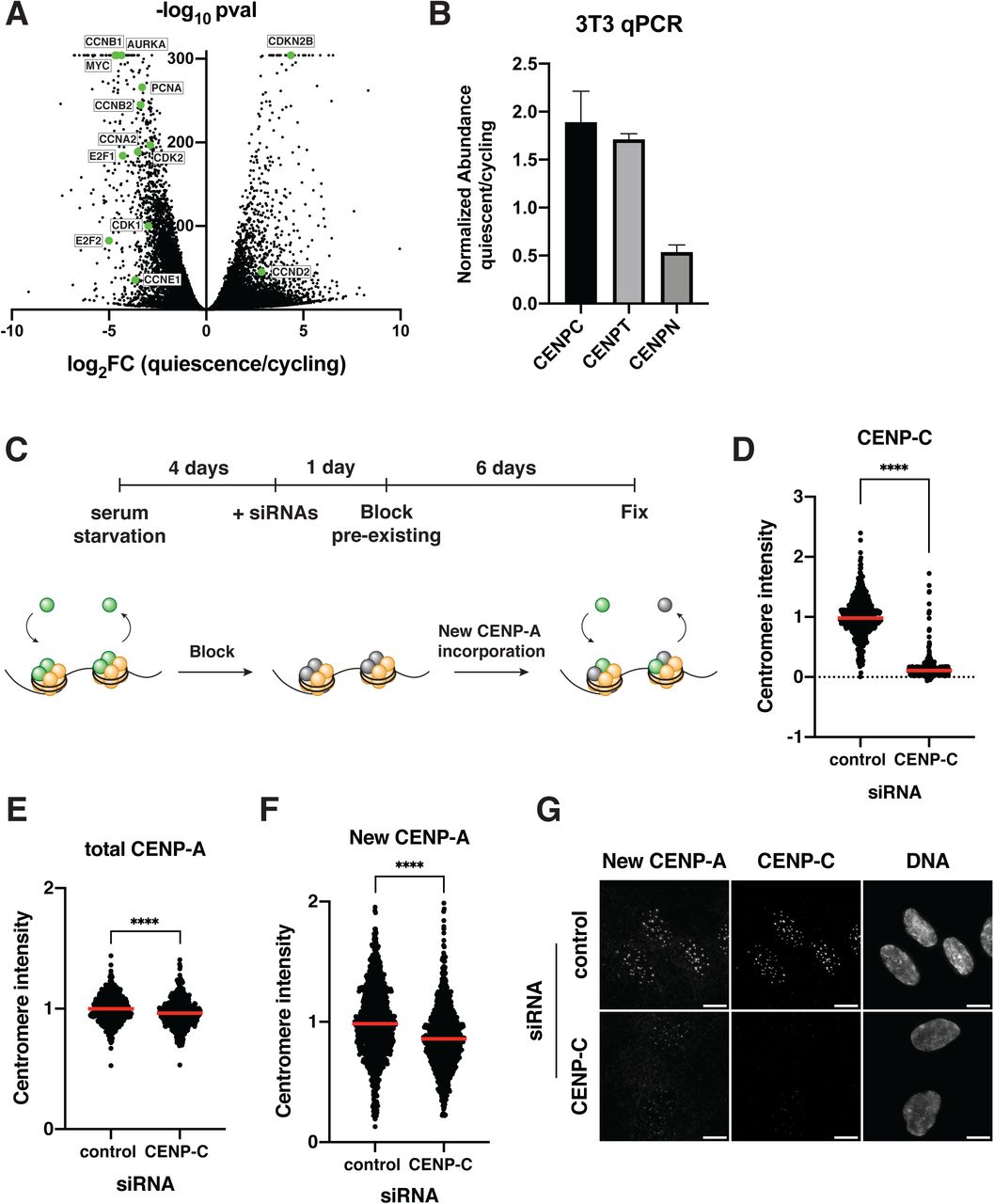

Supplement: figure-S2 — Supplemental Figure 2: CENP-C contributes to CENP-A deposition in quiescent cells A. Volcano plot comparing mRNA abundances in quiescent and cycling RPE1 cells as measured by RNA sequencing. Positive controls, including certain cyclins, cyclin-dependent kinases, and other proliferation factors, are highlighted in green. A p-value cut-off was imposed at p = 6.84E-305 for genes with p-values of 0. Genes with low read counts (total counts < 50) were excluded. B. Graph showing the fold change in mRNA abundance in mouse 3T3 cells between cycling cells and cells in quiescence for 7 days for the indicated centromere component as quantified by qPCR. CT values were normalized to those of GAPDH before comparing quiescent and cycling values. Graph shows at least 3 biological replicates, with 3 technical replicates each for each centromere mRNA. Bars represent mean ± standard deviation. C. Schematic showing experimental design for HaloTag pulse-chase experiments. Unblocked CENP-A is shown in green, blocked CENP-A in gray and other histones in yellow. More experimental details can be found in the methods section. D. Graph showing CENP-C intensity at the centromeres after 7 days of RNAi treatment. Cells were fixed and stained with CENP-C antibody at the end of the experiment from S2C. Each point indicates the average centromere intensity level for all centromeres in a single cell, adjusted for background. Intensity values were normalized to control. Points were aggregated from 3 replicates. **** indicates p<0.0001. n = 486, 432 for control and CENP-C RNAi conditions respectively E. Graph showing total CENP-A intensity at the centromeres after 7 days of RNAi treatment. Cells were fixed and stained with CENP-A antibody at the end of the experiment from S2C. Each point indicates the average centromere intensity level for all centromeres in a single cell, adjusted for background. Intensity values were normalized to control. Points were aggregated from 3 replicates. **** indicates p<0 [file figure-S2.jpg]

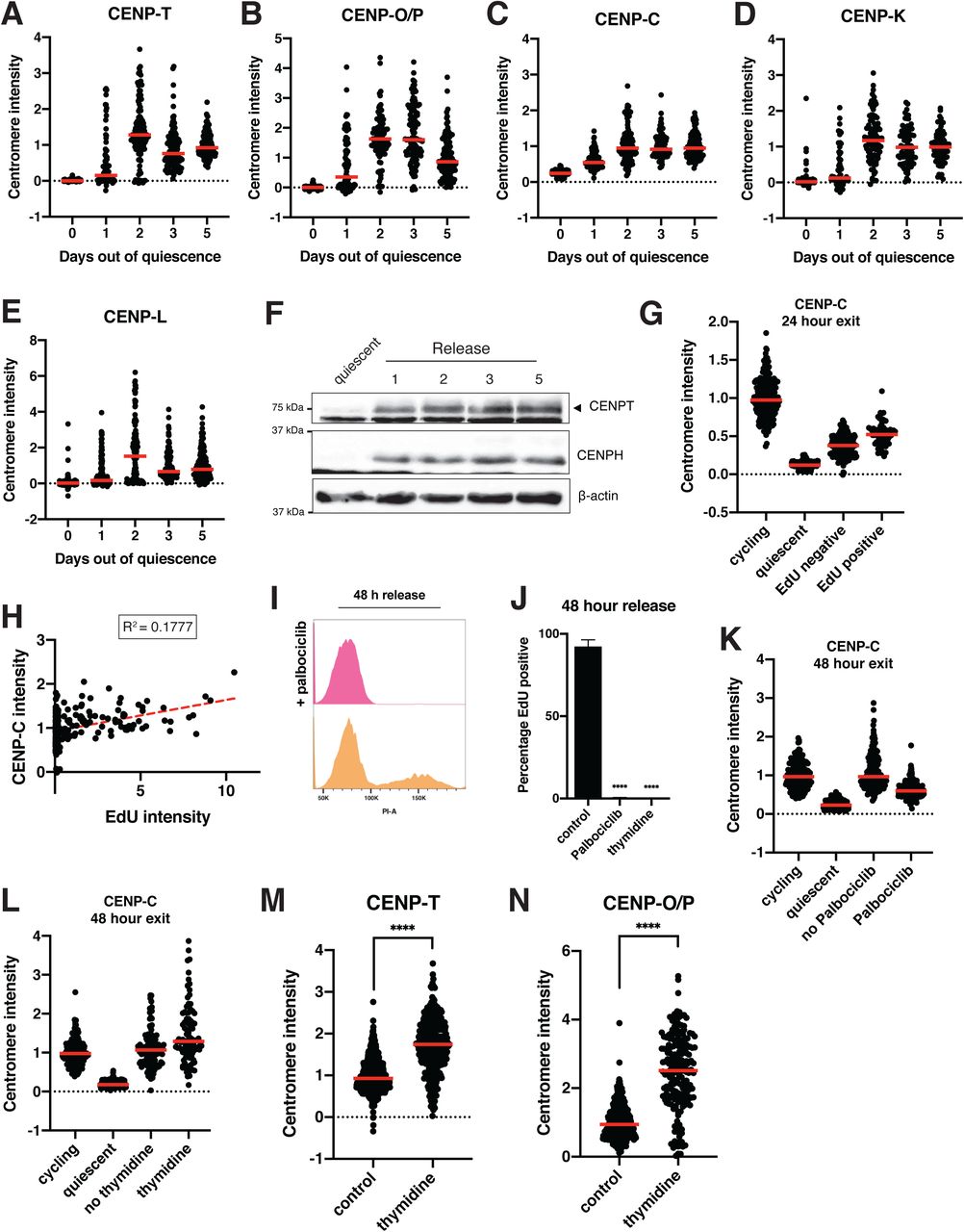

Supplement: figure-S3 — Supplementary Figure 3: The centromere is rapidly reassembled upon cell cycle reentry A. Graph showing CENP-T centromere intensity level over time of quiescence exit. Each point indicates the average centromere intensity level for all centromeres in a single cell, adjusted for background. Intensity values were normalized to day 5. Red line represents the median. Points were aggregated from 2 replicates. n = 232, 94, 116, 115, and 138 cells for 0, 1, 2, 3, and 5-day time points respectively. B. Graph showing CENP-O/P centromere intensity level over time of quiescence exit. Each point indicates the average centromere intensity level for all centromeres in a single cell, adjusted for background. Intensity values were normalized to day 5. Red line represents the median. Points were aggregated from 2 replicates. n = 238, 90, 94, 111, and 110 cells for 0, 1, 2, 3, and 5-day time points respectively. C. Graph showing CENP-C centromere intensity level over time of quiescence exit. Each point indicates the average centromere intensity level for all centromeres in a single cell, adjusted for background. Intensity values were normalized to day 5. Red line represents the median. Points were aggregated from 2 replicates. n = 180, 122, 133, 100, and 144 cells for 0, 1, 2, 3, and 5-day time points respectively. D. Graph showing CENP-K centromere intensity level over time of quiescence exit. Each point indicates the average centromere intensity level for all centromeres in a single cell, adjusted for background. Intensity values were normalized to day 5. Red line represents the median. Points were aggregated from 2 replicates. n = 179, 67, 89, 82, and 80 cells for 0, 1, 2, 3, and 5-day time points respectively. E. Graph showing CENP-L centromere intensity level over time of quiescence exit. Each point indicates the average centromere intensity level for all centromeres in a single cell, adjusted for background. Intensity values were normalized to day 5. Red line represents the medi [file figure-S3.jpg]
